# Anti-TNF certolizumab pegol induces antioxidant response in human monocytes via reverse signaling

**DOI:** 10.1186/s13075-016-0955-8

**Published:** 2016-03-01

**Authors:** Jean Frédéric Boyer, Michel Baron, Arnaud Constantin, Yannick Degboé, Alain Cantagrel, Jean-Luc Davignon

**Affiliations:** Université Paul Sabatier Toulouse III, Toulouse, France; INSERM - CNRS U1043, CPTP, CHU Purpan, 1, Place Baylac, 31300 Toulouse, France; Centre de Rhumatologie, Hopital Pierre Paul Riquet, Toulouse, France

**Keywords:** Transmembrane TNF-α, Reverse signaling, Anti-TNF, Monocytes, Nrf2, Reactive oxygen species, Inflammation, Rheumatoid arthritis

## Abstract

**Background:**

Anti TNF drugs have been widely used in rheumatoid arthritis (RA) but only 70 to 80 % of patients respond to this therapy. Exploring the mode of action of anti-TNF drugs remains important in order to improve the efficiency of the treatment and enhance our knowledge of inflammation. TNF-α exists as classical soluble cytokine as well as transmembrane protein (tmTNF-α). Evidence suggests that tmTNF-α can induce reverse signaling. In the present study, we have explored consequences of reverse signaling in human monocytes using certolizumab pegol (CZP).

**Methods:**

Monocytes were purified from healthy blood donors and were incubated with CZP. Nuclear translocation of Nuclear factor (erythroid-derived 2)-like 2 (Nrf2) was evaluated by wide-field microscopy and cell fractionation. Heme oxygenase 1 (HO-1) was assessed by RT-qPCR and western blot. Monocytes were stimulated with lipopolysaccharide (LPS). IL-1β was quantitated by RT-qPCR. Reactive oxygen species (ROS) were evaluated by flow cytometry using the H_2_DCFDA fluorescent marker.

**Results:**

CZP induced rapid minimal ROS production and Nrf2 nuclear translocation. This was followed by HO-1 mRNA and protein production. IL-1β induction by LPS was inhibited at the mRNA and protein level. At a later time-point, CZP was able to counteract the strong production of ROS induced by LPS.

Reverse signaling was suggested by short kinetics of Nrf2 translocation, extensive washing of CZP and the use of anti-TNF-Rs antibodies.

**Conclusion:**

Our data suggest a novel mechanism of ROS modulation by CZP. This observation sheds new light on the function of reverse signaling and on potential mechanisms of action of anti-TNF drugs.

**Electronic supplementary material:**

The online version of this article (doi:10.1186/s13075-016-0955-8) contains supplementary material, which is available to authorized users.

## Background

Anti-TNF drugs have been widely used in rheumatoid arthritis (RA) for more than 15 years [1]. Only 70–80 % of patients respond to this therapy. To date, there is no explanation for this lack of efficacy [2]. Further understanding of the mode of action of anti-TNF drugs is much needed to improve the efficacy of the treatment and enhance our knowledge of inflammation.

The rationale for anti-TNF therapy in RA is the inhibition of the cytokine cascade, such as IL1, IL6 and granulocyte-macrophage colony-stimulating factor (GM-CSF) [1]. In addition to neutralizing soluble TNF-α, anti-TNF may also induce signal reverse via transmembrane TNF- α (tm TNF-α) [3]. TNF-α, and most of the other members of the TNF superfamily are also transmembrane proteins. Evidence suggests that the membrane-integrated ligands can receive signals and act as receptors, which can transmit feedback signals, called reverse signaling, into the cell [4, 5]. A previous study showed that pre-treatment of monocytes with anti-TNF induces the resistance of monocytes to lipopolysaccharide (LPS) [6]. In monocytes from patients with RA, anti-TNF stimulation was shown to decrease the secretion of inflammatory cytokine and to induce apoptosis [7]. These observations were attributed to reverse signaling. The role of reverse signaling in the initiation and perpetuation of arthritis is not established.

Monocytes are attractive targets for the treatment of RA [8]. Our laboratory is interested in the characterization of the role of tm-TNF-α in monocytes as a possible cell target of anti-TNF, which is now commonly used in the treatment of RA. In a previous study, we investigated the role of TNF-α and adalimumab on CD36 expression in human monocytes. F(ab’)2 fragment obtained from adalimumab induced an increase in CD36 expression due to reverse signaling depending on reactive oxygen species (ROS) [9]. As nuclear factor (erythroid-derived 2)-like 2 (Nrf2) is induced upon ROS production [10–14] and is involved in CD36 induction [15, 16], we hypothesized that it could be induced by anti-TNF. Nrf2 is a redox-sensitive basic leucine zipper transcription factor involved in the transcriptional regulation of many antioxidant and cell protective genes. It plays a role in the control of inflammation. For example, genetic disruption of Nrf2 in different mouse models increases the mortality of mice in response to septic shock, increases the severity of arthritis, and leads to severe allergen-driven airway inflammation and hyper-responsiveness [17, 18].

HO-1 is an enzyme regulated by Nrf2 [19]. In inflammation, heme oxygenase 1 (HO-1) protects cells from oxidative damage during stress [20]. In a model of genetic inactivation of HO-1, mice are vulnerable to mortality and hepatic necrosis when challenged with endotoxin [21]. In a human case of HO-1 deficiency the patient presented with growth retardation, anemia, iron deposition, and vulnerability to stressful injury [22]. Endothelial cells from mice with HO-1 knockout (KO) have enhanced sensitivity to complement [23]. Anti-inflammatory effects of HO-1 usually result from the degradation of pro-inflammatory free heme, and the production of the anti-inflammatory compounds as carbon monoxide (CO), biliverdin/bilirubin and free iron [24]. HO-1 could thus be a therapeutic target in RA.

In this work we thus used certolizumab pegol (CZP) to investigate the induction of Nrf2 through tmTNF-α. CZP was chosen because it is a monovalent Fab anti-TNF antibody linked with a polyethylene glycol (PEG) fragment that has no Fc fragment [7, 25]. We showed that CZP induced rapid ROS induction and nuclear translocation of Nrf2 and expression of HO-1. Moreover, LPS-induced ROS expression and IL-1 production were attenuated by CZP, suggesting an effect on toll-like receptor (TLR)4-induced inflammation. Our data point to a mechanism, which, due to reverse signaling, may regulate inflammation.

## Methods

### Reagents

LPS from *Eschericia coli* 055:B5 was purchased from Sigma (St Louis, MO, USA). Nrf2 (H300) antibodies were purchased from Santa Cruz Biotechnology (Dallas, TX, USA), anti-rabbit IgG horse radish peroxidase (HRP)-linked antibodies, Alexa Fluor 488 F(ab’)2 anti-rabbit IgG, tatabox binding protein (TBP) and HO-1 (P249) antibodies were purchased from Cell Signaling Technology (Danvers, MA, USA) and glyceraldehyde 3-phosphate dehydrogenase (GAPDH) (ABS16) antibodies were purchased from Millipore (Temecula, CA, USA). Blocking antibodies against TNF receptor 1 (TNFR1) (clone #16805) and TNFR2 (clone #22210) were purchased from R&D Systems (Minneapolis, MN, USA). Anti-CD14 (HCD14) antibody was from Biolegend, San Diego, CA, USA. H_2_DCFDA (C-2938) was purchased from Life Technologies (Carlsbad, CA, USA). The 4',6-diamidino-2-phenylindole (DAPI) was purchased from Biolegend (San Diego, CA, USA). The diphenyleneiodonium chloride (DPI) and wortmannin inhibitors were purchased from Sigma.

### Preparation of peripheral blood mononuclear cells (PBMC) and monocyte purification

PBMC were isolated from healthy blood donors (Etablissement Français du Sang Transfusion Center, Toulouse, France). Ethical approval was obtained from the Transfusion Center. Informed consent was obtained from blood donors. PBMC were separated by ficoll density gradient centrifugation at 700 g for 20 minutes. They were then resuspended in PBS 2 mM ethylenediaminetetraacetic acid (EDTA) and thoroughly washed (centrifugation at 350 g for 8 minutes six times). The percentage of monocytes was evaluated by flow cytometry on a FACSCalibur cytometer (BD Bioscience, San Diego, CA, USA) with an anti-CD14 (HCD14) antibody (Biolegend, San Diego, CA, USA)). CD14+ monocytes were purified by negative immunomagnetic bead separation using Dynabeads Untouched Human Monocytes kit (Life Technologies, Carlsbad, CA, USA). Purity of CD14+ monocytes was >90 %. Monocytes were then incubated in Macrophage SFM medium (Life Technologies) and plated one hour before applying any treatment. Cells were incubated with CZP (5 μg/ml) for the indicated times, then thoroughly washed as described [6, 25].

### Quantitative real-time PCR

Total RNA from 4 × 10^6^ monocytes was isolated using High Pure RNA Isolation Kit (Roche Diagnostics GmbH, Mannheim, Germany) and complementary DNA (cDNA) synthesized with RevertAid Minus Reverse Transcriptase (Thermo Fisher Scientific, Waltham, MA, USA). Gene expression was performed using LightCycler 480 SYBR Green Master Mix and LightCycler 480 System instrument (Roche Diagnostics GmbH). All primers were designed using ProbeFinder Software (Roche Applied Science website), and synthetized by Sigma Life Science (St Quentin Fallavier, France) (Table 1).

**Table 1:** Primers sequences used for qPCR

Gene	Accession number	Forward primer	Reverse primer
*gapdh*	J04038.1	cagcctcaagatcatcagca	gtcttctgggtggcagtgat
*hmox1*	NM_002133.2	ggcagagggtgatagaagagg	agctcctgcaactcctcaaa
*IL1B*	NM_000576.2	aaagcttggtgatgtctggtc	ggacatggagaacaccacttg

### ROS measurements

For intracellular ROS measurement, we used the H_2_DCFDA compound. Monocytes (4 × 10^5^ cells) were grown in a 24 well-plate in 0.5 ml Macrophage SFM medium. All treatment conditions were performed in duplicate. Monocytes were incubated, or not, for 5, 15 and 30 minutes with 5 μg/ml of CZP. Then, culture medium was discarded and replaced by PBS containing 2 μM H_2_DCFDA. Monocytes were incubated for 30 minutes and then detached from the well in 300 μl of PBS 5 mM EDTA at 4 °C. The level of fluorescence (geometric mean) was measured by flow cytometry on a FACSCalibur cytometer.

In another experiment, monocytes were incubated, or not, for 1 h with 5 μg/ml of CZP, washed three times with medium to remove unfixed CZP, and then incubated for 18 h in medium containing LPS. The ROS production was then measured. All cytometry data were analyzed by FlowJo (TreeStar Inc, Ashland, OR, USA).

### Western blot analysis

Total extracts from 4 × 10^6^ monocytes lysed in 50 μl of Laemmli buffer were denatured at 95 °C for 10 minutes and sonicated: 15–20 μl were run on Novex NuPAGE 4–12 % Bis-Tris mini gels and transferred on nitrocellulose membrane with X-Cell blot module (Life Technologies). After incubation with primary and secondary HRP-coupled antibodies, labeled proteins were visualized by enhanced chemiluminescence with ECL Prime Western Blotting Detection Reagent (GE Healthcare, Piscataway, NJ, USA) on a ChemiDoc XRS+ imaging system (Bio-Rad Laboratories, Hercules, CA, USA). All images were analyzed with the Image Lab 5.0 software (Bio-Rad). Antibodies to GAPDH and TBP were used to normalize the loading quantities of protein in the different lanes of the gels.

### Preparation of nuclear extracts

Monocytes (4–5 × 10^6^) grown in 6-well plates and 2 ml of Macrophage SFM medium were scraped in 1 ml of ice-cold PBS supplemented by protease inhibitor cocktail (PIC, P8340, Sigma) at 1:100 dilution and harvested at 800 g and 4 °C for 4 minutes. Cells were resuspended in 50 μl of lysis buffer (10 mM TRIS pH 7.3, 1 mM MgCl2, 250 mM sucrose, 0.25 % NP40, 1:50 PIC) and left on ice for 10 minutes. After centrifugation at 1500 g for 4 minutes at 4 °C, the nuclear enriched pellet was lysed in 50 μl of Laemmli buffer. The lysates were denatured for 8 minutes at 95 °C and sonicated before western blot analysis.

### Immunofluorescence microscopy

Monocytes (3 × 10^5^ cells) in 0.5 ml of Macrophage SFM medium were plated for 1 h on a glass coverslip in a 24-well plate and incubated, or not, by 5 μg/ml CZP. After 30 minutes, the medium was removed by aspiration and cells were fixed and permeabilized with 250 μl methanol at 80 % over 4 minutes at –20 °C. After washing with PBS and PBS 0.25 % BSA, cells were incubated with the Nrf2 (H300) antibody at 1:250 dilution for 1 h. Cells were rinsed three times with PBS-BSA and then incubated with secondary anti-rabbit AF488 antibody for another 1 h. DAPI at a final concentration of 0.3 μM was added for 10 minutes and cells were washed another three times with PBS-BSA. The coverslip glass was mounted on a microscopic slide with Mowiol mounting medium. All observations were made on an Apotome Zeiss device equipped with a × 63 objective. Images were obtained using Zen software (Carl Zeiss Microscopy GmbH, Jena, Germany).

### Statistics

All experiments were performed more than three times. The values are expressed as the mean ± standard deviation (SD). The *t* test was used to assess the significance of differences between two conditions. All *p* values are two-sided, and *p* values equal to or below 0.05 were considered significant.

## Results

### Anti-TNF induces ROS production and nuclear translocation of Nrf2 translocation in human monocytes

**Fig. 1:**
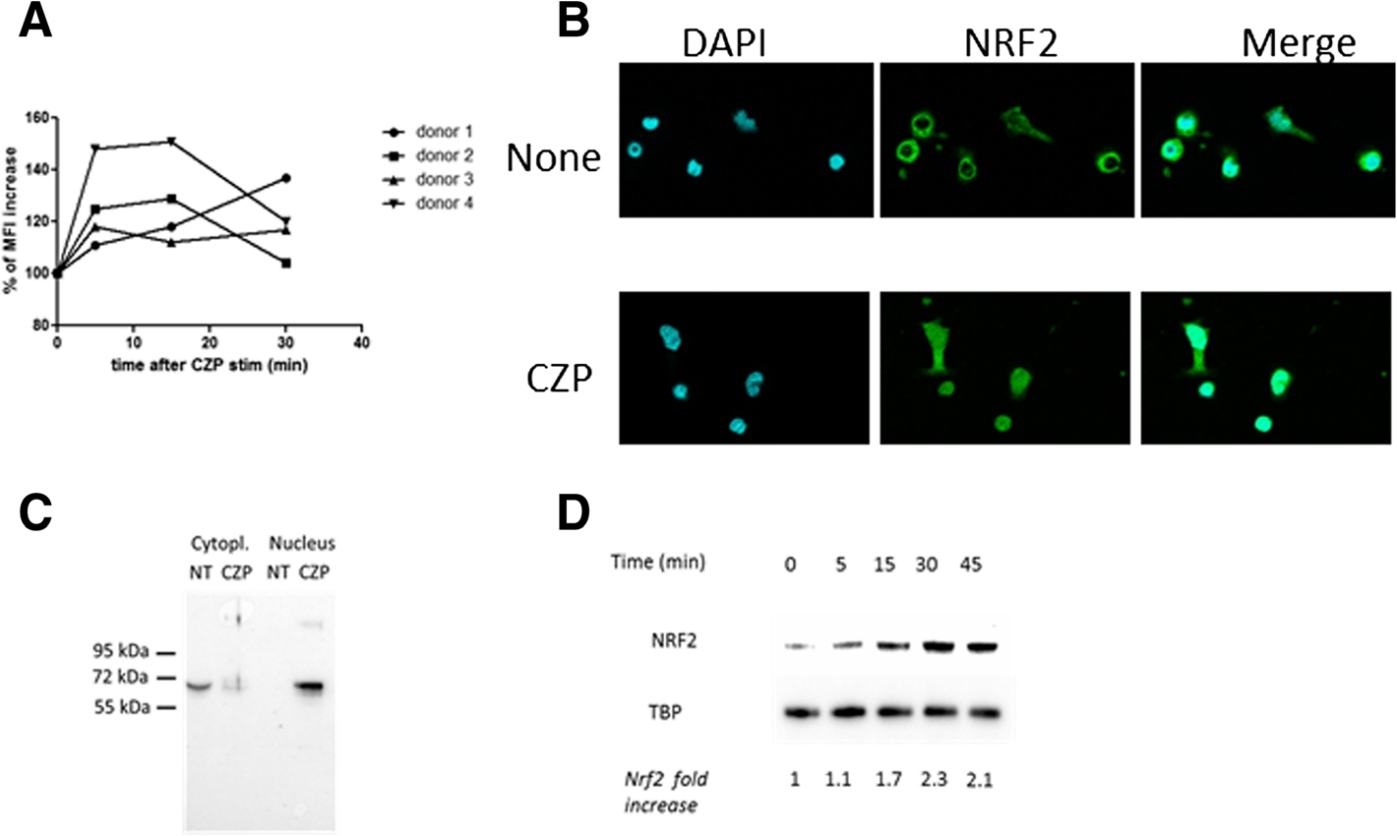
Certolizumab pegol (*CZP*) induces rapid production of reactive oxygen species (ROS) and nuclear translocation of nuclear factor (erythroid-derived 2)-like (*Nrf2*). Monocytes were incubated, or not, with CZP (5 μg/ml) and ROS production was assessed by H2DCFCA using flow cytometry. The time course of rapid ROS production over 30 minutes is shown in monocytes from four different healthy donors (**a**). Monocytes were incubated, or not, with CZP (5 μg/ml) for 90 minutes, and cellular localization of Nrf2 was analyzed by wide-field microscopy using an anti-Nrf2 antibody. 4',6-diamidino-2-phenylindole (*DAPI*) was used as control for nucleus staining (**b**). Cytoplasmic and nuclear extracts were prepared after 90 minutes of cell culture and analyzed for Nrf2 detection (**c**). Time course analysis of Nrf2 translocation in nuclear fraction-enriched cell extracts of monocytes incubated, or not, with CZP (5 µg/ml) (**d**). TATA box binding protein (*TBP*) was used as control (**d**). Data are representative of four experiments (**b**, **c**, **d**). *MFI* geometric mean of fluorescence intensity

In a previous report, we observed that the increase in CD36 expression induced by the anti-TNF agent adalimumab was dependent on nicotinamide adenine dinucleotide phosphate-oxidase (NADPH) activation [9]. We showed that CZP induces cytoplasmic ROS production shortly after incubation (within 5–30 minutes) with monocytes (Fig. 1a). As Nrf2 is involved in the response to ROS and is involved in the regulation of CD36 expression [16], we assessed its nuclear translocation in monocytes. As shown in Fig. 1b, the anti-TNF CZP induced the nuclear translocation of Nrf2. Nuclear translocation was also confirmed by western blot of cellular and nuclear extracts (Fig. 1c). Expression of Nrf2 in the nucleus increased rapidly after incubation with anti-TNF (Fig. 1d).

### Anti-TNF induces HO-1

**Fig. 2:**
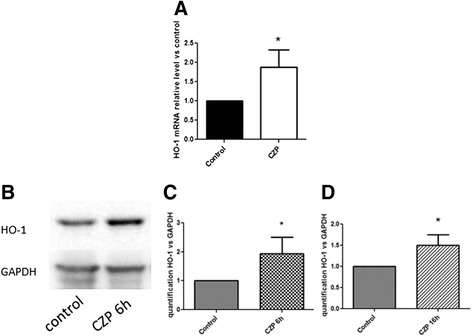
Certolizumab pegol (*CZP*) induces heme oxygenase 1 (*HO-1*) expression. Monocytes were incubated, or not, with CZP (5 μg/ml) for 4 h of culture. RNA was isolated and qRT-PCR was performed to quantify HO-1 mRNA. Quantification of five experiments. *Paired *t* test <0.05 (**a**). Monocytes were incubated, or not, with CZP (5 μg/ml) during 6 or 16 h of culture. HO-1 protein was assessed by western blot (**b**). Quantification of five experiments was performed at 6h (**c**) and at 16h (**d**). Paired *t* test <0.05. *GAPDH* glyceraldehyde 3-phosphate dehydrogenase

To further illustrate the functionality of the targeting of Nrf2 by anti-TNF we assessed the messenger RNA (mRNA) production of HO-1, a key enzyme implicated in heme degradation and regulated by Nrf2 [19]. As shown in Fig. 2a, CZP induced a twofold increase of HO-1 mRNA after 4 h of cell culture. A similar increase in HO-1 protein was also observed after 6 and 16 h of cell culture (Fig. 2b-d).

### Induction of HO-1 by anti-TNF is dependent upon ROS production and PI3 kinase activity

**Fig. 3:**
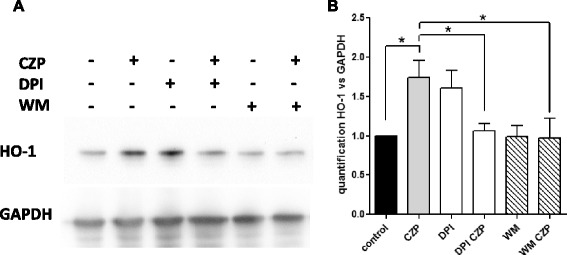
Roles of nicotinamide adenine dinucleotide phosphate-oxidase (NADPH) and Pi3 kinase in the induction of heme oxygenase 1 (*HO-1*) by certolizumab pegol (*CZP*). Monocytes were incubated, or not, with CZP 5 μg/ml in the presence or not of diphenyleneiodonium chloride (*DPI*) (10 μM) and wortmannin (0.4 μM) over 16 h. HO-1 was assessed by western blot. One representative blot is shown (**a**). Quantification of three western blot experiments was performed. *Paired *t* test <0.05 (**b**). *GAPDH* glyceraldehyde 3-phosphate dehydrogenase, *WM* Wortmannin

Nicotinamide adenine dinucleotide phosphate-oxidase [NADPH] is a major source of ROS [26] which, as a source of stress, are involved in the activation of Nrf2 [11–13]. To test if NADPH was implicated in HO-1 induced by anti-TNF, we used an inhibitor of ROS production induced by NADPH, DPI. Figure 3a and b show that DPI used alone induced ROS on its own, as already described [27]. However, DPI consistently inhibited the induction of HO-1 observed with CZP, indicating a role for NADPH in the induction of HO-1. PI3k is activated by calcium flux and has been reported to activate NADPH oxydase [28]. As shown in Fig. 3a and b, wortmannin, a PI3K inhibitor, completely blocked the induction of HO-1 observed with anti-TNF CZP. This suggests that PI3K is involved in the induction of HO-1 by CZP.

### Induction of HO-1 is not due to TNFR and results from reverse signaling

**Fig. 4:**
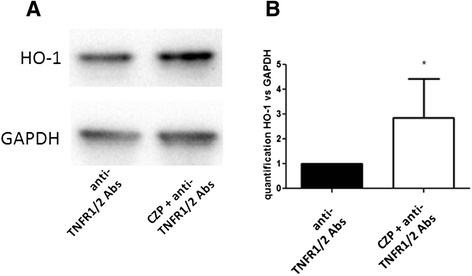
Heme oxygenase 1 (*HO-1*) induction by anti-TNF certolizumab pegol (*CZP*) is due to reverse signaling. Monocytes were incubated, or not, with CZP (5 μg/ml) for 16 h in the presence of TNF receptor 1 (*TNFR1*) and TNFR2 (5 μg/ml) blocking antibody. HO-1 protein expression was assessed by western blot (**a**). Quantification of four western blot experiments was performed. Paired t test *p* = 0.05 (**b**). *GAPDH* glyceraldehyde 3-phosphate dehydrogenase, *Abs* antibodies

Induction of HO-1 may result from blocking the interaction of TNF-α with its receptors. To elucidate whether the induction of HO-1 by anti-TNF results from a reverse signaling phenomenon we inhibited the TNFR1 and TNFR2 by specific blocking antibodies. As shown in Fig. 4, the anti-TNF-induced increase in HO-1 expression was still observed in the presence of the anti-TNFR1 and TNFR2 blocking antibody. This suggests that the induction of HO-1 by CZP was not mediated by TNFR and thus, resulted from reverse signaling.

### CZP inhibits LPS-induced IL-1β and ROS production

**Fig. 5:**
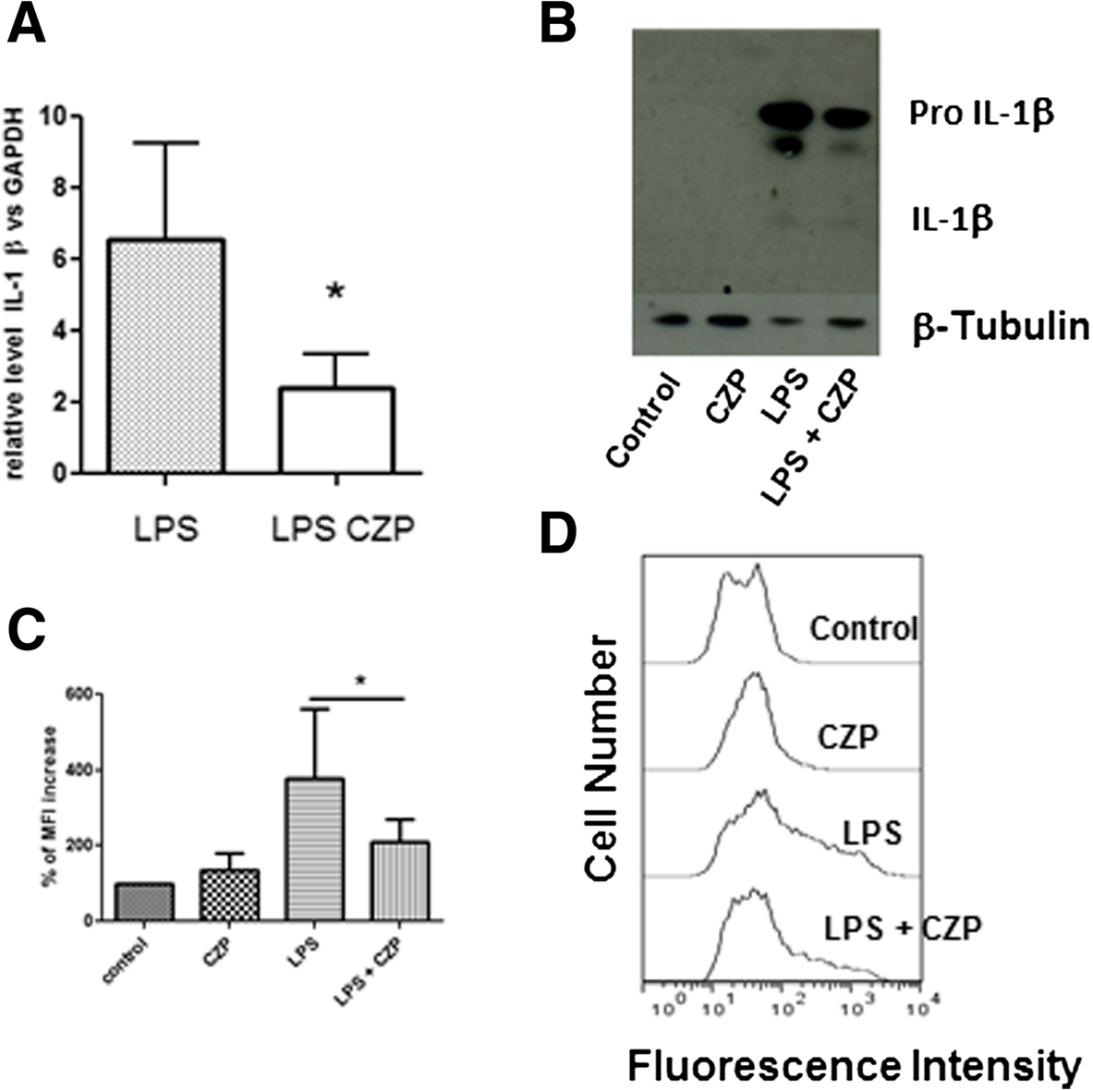
Certolizumab pegol (*CZP*) directly induces inhibition of the toll-like receptor 4 pathway. Expression of IL1β: monocytes were incubated, or not, with CZP (5 μg/ml) for 1 h, washed, then stimulated with lipopolysaccharide (*LPS*) (10 ng/ml) for 16 h. IL1β mRNA was assessed by qRT-PCR. Results are expressed as relative level of IL1β mRNA mean ± SD in three experiments (**a**). IL1β protein was assessed by western blot in monocytes stimulated as in (**a**) for 16 h (**b**). Data shown are representative of three experiments. Monocytes were incubated, or not, with CZP (5 μg/ml) for 1 h, washed and stimulated with LPS (10 ng/ml) for 16 h. The level of ROS production was expressed as the percentage of variation of the geometric mean fluorescence intensity (*MFI*) of H2DFCDA as analyzed by flow cytometry (**c**) in five experiments. *Paired *t* test <0.05 for comparison of LPS and LPS + CZP. A representative cytometry experiment in shown (**d**). *GAPDH* glyceraldehyde 3-phosphate dehydrogenase

Previous studies have shown that anti-TNF decreases the sensitivity of TLR signaling in monocytes [6, 29]. The mechanism has not been reported in detail. As HO-1 and Nrf2 are reported to be involved in the regulation of TLR4 signaling [30, 31], we investigated the impact of anti-TNF on TLR-4-induced ROS production. In a first step, we validated the repression of LPS-induced IL1β by anti-TNF. After short incubation of monocytes with CZP, cells were extensively washed and analyzed. As shown in Fig. 5, CZP induced a decrease in LPS-induced IL1β production at the mRNA (Fig. 5a) and protein (Fig. 5b), levels. This suggested that CZP modulated the TLR4-mediated inflammatory signal. We then evaluated the modulation of LPS-induced ROS by CZP. We observed (Fig. 5c) that pre-treatment of monocytes with CZP, significantly inhibited the ROS production induced by TLR4 stimulation with LPS. A representative cytometry experiment is shown in Fig. 5d. This suggests that engaging tmTNF-α modified the monocyte response to LPS.

## Discussion

In this work, we showed that anti-TNF CZP induced rapid ROS production and subsequent nuclear translocation of Nrf2 in human monocytes. As a consequence, HO-1 expression was increased. Moreover, LPS-induced IL1ß and ROS production were inhibited by CZP. To our knowledge, this is the first description of Nrf2 activation and ROS modulation by anti-TNF via reverse signaling.

Reverse signaling has been characterized on the basis of several factors. First, induction of HO-1 was still observed in the presence of specific anti-TNF receptors 1 and 2 blocking antibodies and thus, did not result from TNF-α neutralization by CZP. Second, TNF-α itself is known to induce Nrf2 [32]. If our results were due to mere neutralization of soluble TNF, results opposite to ours would have rather been observed on HO-1 and Nrf2. Third, the kinetics of the induction of ROS were rapid and inhibition of TLR signaling after washing away the anti-TNF was still observed. A more direct approach would probably be to test monocytes with knocked down TNFR1/2 and with expression of non-cleavable TNF. However, we observed that these cells express high levels of Nrf2 when transfected with small interfering RNA (siRNA) or infected with viruses (our unpublished data), making this approach difficult.

The mechanism leading to Nrf2 activation is likely to be due to the relatively small amount of induced ROS as early as 5 minutes after stimulation with anti-TNF (10–50 % increase). Our data suggest that reverse signaling in monocytes is an immediate or early event that controls subsequent monocyte activation. LPS stimulation induced a much stronger increase (400 %) in ROS that was potently reduced by CZP in a 16-h timeframe. Moreover, CZP, like adalimumab [9], induced CD36 expression (see Additional file 1). Altogether, the induction of CD36, the resistance to LPS and the rapid induction of Nrf2 by anti-TNF may represent features of macrophage polarization to an anti-inflammatory activation phenotype. As a comparison, IL4 has been shown to deliver a signal that counteracts LPS-induced polarization and induces CD36 [33, 34]. Future experiments will determinethe consequences of reverse signaling induced by anti-TNF on monocyte polarization.

Besides playing a role in the removal of ROS-induced electrophiles and protection of cells from injuries, Nrf2 play a role in the control of inflammation [35]. In a mouse model of colitis, inflammation was increased in the colonic tissues of Nrf2-/- mice compared with their wild-type Nrf2+/+ counterparts [36]. Nrf2 is activated in the joints of arthritic mice and of patients with RA [18]. Data exploring the existence of a link between response to anti-TNF and induction of Nrf2 in RA are needed.

Induction of HO-1 in arthritis has been the topic of a large number of publications. A study reported that infliximab, an anti-TNF agent, increased HO-1 expression in human monocytes from patients with RA [37]. The authors suggest that it resulted from the inhibition of the downregulation of HO-1 by TNF-α. However, the persistence of HO-1 induction with anti-TNF-R blocking antibodies observed in our study suggests an active mechanism of anti-TNF in HO-1 induction [32]. At the cellular level, HO-1 decreases the secretion of metalloproteases by chondrocytes [38]. It has also been shown to inhibit phospholipase A2 induced by TNF-α in synovial fibroblasts in RA [39] and has been reported to favor protection of the endothelial cells to complement injury [23]. Thus, HO-1 may be related to the resolution of inflammation and arthritis.

An important result of this work appears to be the inhibition of the TLR pathway reverse signaling induced by anti-TNF. Such inhibition has previously been reported, but no clear mechanism was proposed [5]. Our present data on the decreased IL-1β production by CZP confirm those published by Nesbitt et al. and further show that inhibition occurs at the level of specific mRNA production [25]. A recent publication reported that IL-6 is also inhibited in mouse macrophages [40]. This needs to be explored in human macrophages.

## Conclusions

**Fig. 6:**
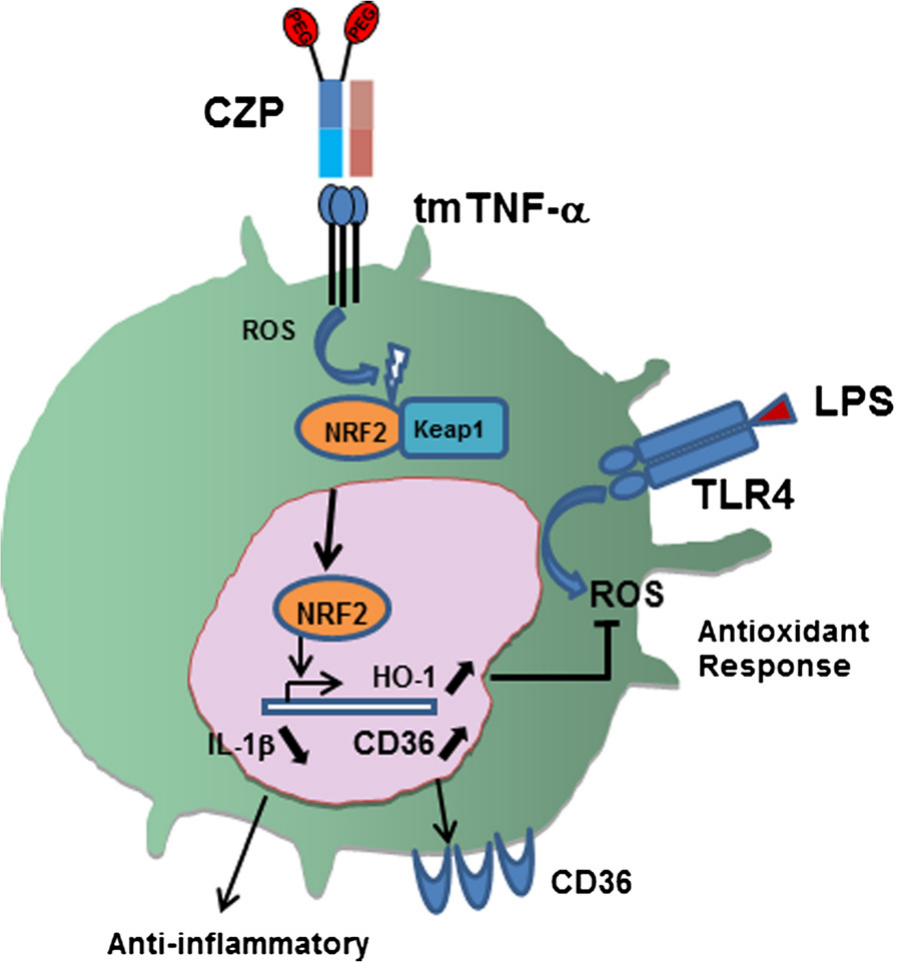
Schematic representation of certolizumab pegol (*CZP*) action on transmembrane TNF (*tmTN*F) and subsequent regulation. The Anti-TNF agent CZP induces rapid reactive oxygen species (*ROS*) production through its binding to tmTNF-α. Nuclear factor (erythroid-derived 2)-like 2 (Nrf2) is translocated to the nucleus. Heme oxygenase 1 (HO-1) and CD36 are induced. The toll-like receptor 4 (*TLR4*) ligand lipopolysaccharide (*LPS*), which mimics some of the triggers of rheumatoid arthritis (RA), induces strong production of ROS and IL-1β, which are both inhibited by pretreatment with CZP

Figure 6 depicts our data and our current view of reverse signaling induced by CZP. Our current study describes antioxidant response induced by anti-TNF-induced reverse signaling in monocytes. Induction of Nrf2 and HO-1, and decrease of LPS-induced ROS and IL-1β in monocytes, may contribute to the therapeutic effects of anti-TNF agents in inflammatory conditions.

## Additional file

Additional file 1: Induction of CD36 by certolizumab pegol (CZP). Human monocytes were incubated for 24 hours with 5 μg/ml CZP. CD36 expression was evaluated by flow cytometry using anti-CD36-PE antibody. Summary of five independent experiments: *p* <0.01. *MFI* geometric mean of fluorescence intensity. (TIF 97 kb)

## Abbreviations

BSA: bovine serum albumin
cDNA: complementary DNA
CZP: certolizumab pegol
DAPI: 4',6-diamidino-2-phenylindole
DPI: diphenyleneiodonium chloride
EDTA: ethylenediaminetetraacetic acid
GM-CSF: granulocyte-macrophage colony-stimulating factor
HO-1: heme oxygenase 1
HRP: horse radish peroxidase
GAPDH: glyceraldehyde 3-phosphate dehydrogenase
IL: interleukin
LPS: lipopolysaccharide
mRNA: messenger RNA
NADPH: nicotinamide adenine dinucleotide phosphate-oxidase
Nrf2: nuclear factor (erythroid-derived 2)-like 2
PBMC: peripheral blood mononuclear cells
PBS: phosphate-buffered saline
PEG: polyethylene glycol
RA: rheumatoid arthritis
ROS: reactive oxygen species
siRNA: small interfering RNA
TBP: TATA box binding protein
TLR: toll-like receptor
tmTNF-α: transmembrane tumor necrosis factor-α
TNF: tumor necrosis factor
TNFR: tumor necrosis factor receptor
