# Supplementary figures and images for: Anti-TNF certolizumab pegol induces antioxidant response in human monocytes via reverse signaling
Source: Arthritis Res Ther. 2016 Mar 1;18:56. doi: 10.1186/s13075-016-0955-8 (PMC4774095; doi:10.1186/s13075-016-0955-8)

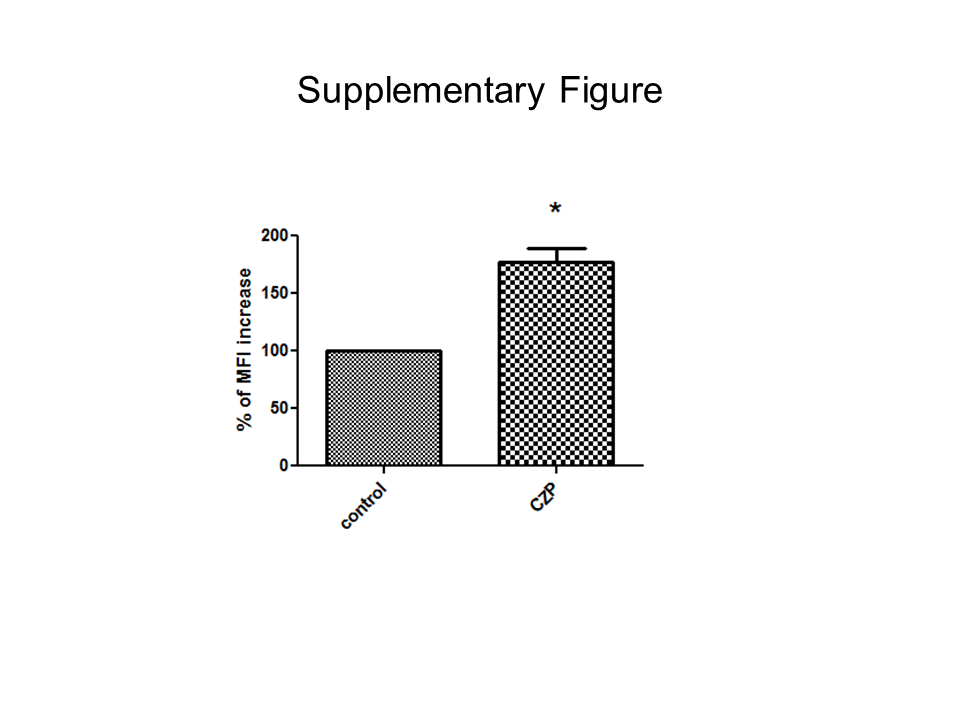

Supplement: Additional file 1: — Induction of CD36 by certolizumab pegol (CZP). Human monocytes were incubated for 24 hours with 5 μg/ml CZP. CD36 expression was evaluated by flow cytometry using anti-CD36-PE antibody. Summary of five independent experiments: p <0.01. MFI geometric mean of fluorescence intensity. (TIF 97 kb) [file 13075_2016_955_MOESM1_ESM.tif]
